# Minimal Residual Disease Assessment Within the Bone Marrow of Multiple Myeloma: A Review of Caveats, Clinical Significance and Future Perspectives

**DOI:** 10.3389/fonc.2019.00699

**Published:** 2019-08-20

**Authors:** Alessandra Romano, Giuseppe Alberto Palumbo, Nunziatina Laura Parrinello, Concetta Conticello, Marina Martello, Carolina Terragna

**Affiliations:** ^1^Department of Surgery and Medical Specialties, University of Catania, Catania, Italy; ^2^Division of Hematology, Azienda Ospedaliero-Universitaria Policlinico Vittorio Emanuele di Catania, Catania, Italy; ^3^Dipartimento di Scienze Mediche, Chirurgiche e Tecnologie avanzate “G.F. Ingrassia,” University of Catania, Catania, Italy; ^4^Dipartimento di Medicina Specialistica, Diagnostica e Sperimentale (DIMES), Università degli Studi di Bologna, Bologna, Italy; ^5^Istituto di Ematologia “L.A.Seràgnoli,” Azienda Ospedaliera Sant'Orsola-Malpighi, Bologna, Italy

**Keywords:** multiple myeloma, flow cytometry, NGS, liquid biopsy, minimal residual disease

## Abstract

There is an increasing clinical interest in the measure and achievement of minimal residual disease (MRD) negativity in the bone marrow of Multiple Myeloma (MM) patients, as defined equally either by Multicolor Flow Cytometry (MFC) or by Next Generation Sequencing (NGS) technologies. At present, modern technologies allow to detect up to one on 104 or on 105 or even on 106 cells, depending on their throughput. MFC approaches, which have been progressively improved up to the so-called Next Generation Flow (NGF), and NGS, which proved clear advantages over ASO-PCR, can detect very low levels of residual disease in the BM. These methods are actually almost superimposable, in terms of MRD detection power, supporting the lack of unanimous preference for either technique on basis of local availability. However, some technical issues are still open: the optimal assay to use to detect either phenotype (e.g., next generation multidimensional flow cytometry, imaging) or genotype aberrations (e.g., ASO-RQ PCR, digital droplet PCR, NGS) and their standardization, the sample source (BM or peripheral blood, PB) and its pre-processing (red-cell lysis vs. Ficoll, fresh vs. frozen samples, requirement of CD138+ cells enrichment). Overall, MRD negativity is considered as the most powerful predictor of favorable long-term outcomes in MM and is likely to represent the major driver of treatment strategies in the near future. In this manuscript, we reviewed the main pitfalls and caveats of MRD detection within bone marrow in MM patients after front-line therapy, highlighting the improving of the currently employed technology and describing alternative methods for MRD testing in MM, such as liquid biopsy.

## Introduction

In Multiple Myeloma (MM), the clonal neoplastic plasma cells (PCs) grow within a microenvironment niche (1, 2), which provide factors promoting their longevity, either within or out of bone marrow (BM) (1–3). Since PC-niches can be localized within the BM, or, less frequently, in the spleen, liver, mucosa-associated tissues or chronically inflamed tissues (4). MM has spatial (5) and temporal heterogeneity (6), with a plethora of sub-clonal mutations carried only by a fraction of the tumor PCs (7), associated with variable clinical outcomes and responsible for different evolutions of intramedullary and extramedullary disease. Therefore, patients might achieve and maintain a complete serological response, with active and proliferating plasmacytomas (8).

The clinical course of MM is characterized by the appearance of a monoclonal protein in serum and/or urine and symptomatic organ damage such as renal impairment, osteolytic bone lesions, hypercalcemia, anemia and recurrent infections, together with high involved/uninvolved serum κ/λ ratio ≥100, more than one focal lesion on magnetic resonance imaging (MRI), or ≥60% clonal plasma cells (9).

After treatment, the depth of response is clinically relevant in all settings of MM patients: newly diagnosed, either eligible (10) or not (11) to autologous stem cell transplantation, and relapsed/refractory (12); nevertheless, not all patients require deep response for long-term control of disease (13).

**Figure 1:**
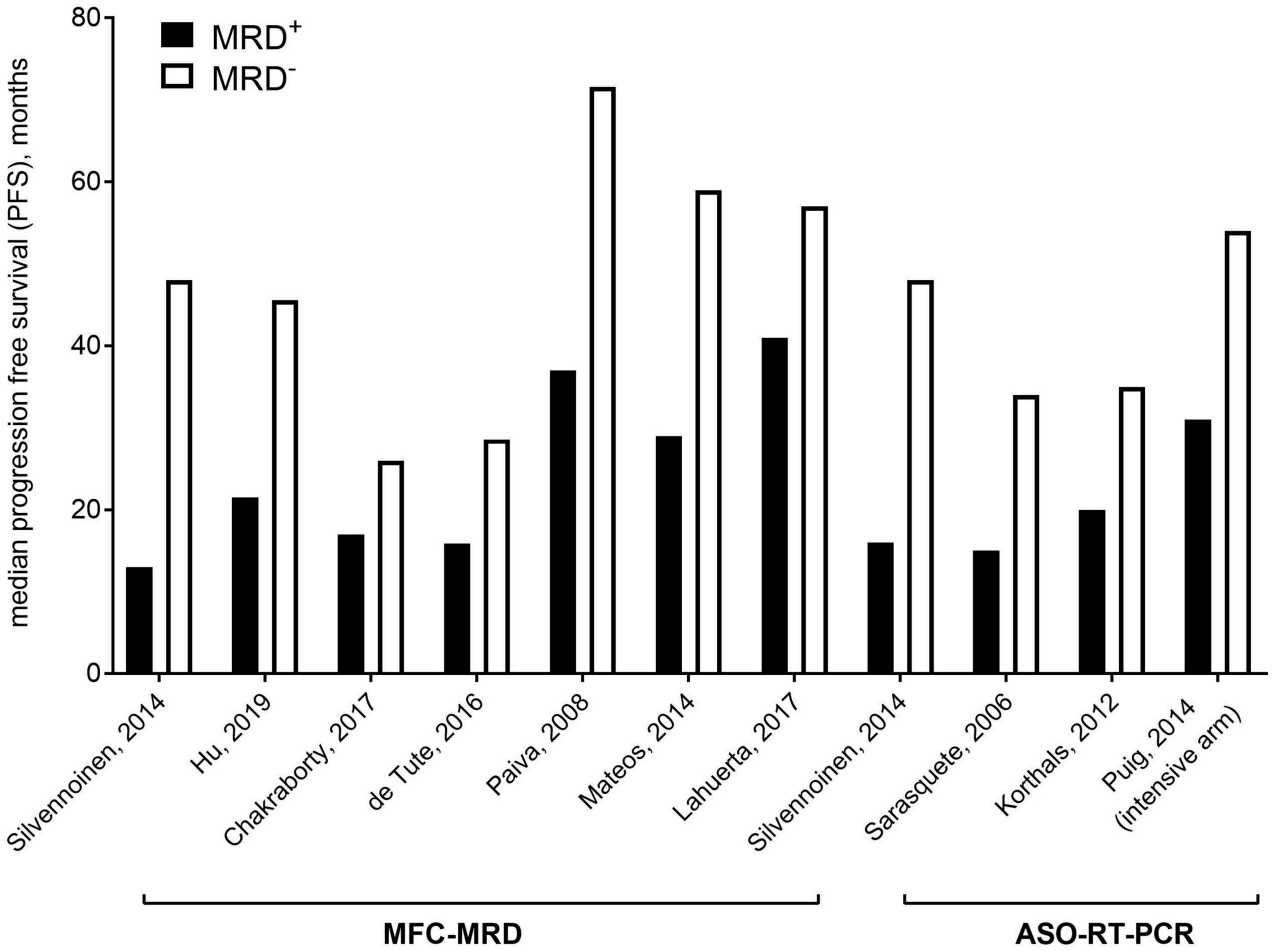
Median progression free survival based on MFC/ASO-qRT-PCR MRD in selected studies.

Recently, the International Myeloma Working Group (IMWG) has defined a revised criteria of responses for patients with MM, by including minimal residual disease (MRD) (9) (Table 1); indeed, several studies (14–30) (Figure 1), also confirmed by two meta-analysis (16, 30), consistently showed inferior outcomes in patients remaining MRD-positive, despite the achievement of complete remission (CR). Data pooled from 5 studies involving 574 patients showed that MRD negativity was superior to CR for survival prediction, with median progression free survival (PFS) of 56 months (HR 0.44 (95% CI 0.34–0.56) and median overall survival (OS) of 112 months (HR 0.47 (95% CI 0.33–0.67) (16).

**Table 1:** IMWG criteria of response for patients with MM.

**Response subCategory**	**Response criteria**
Sustained MRD-negative	MRD negativity in the marrow (NGF or NGS, or both) and by imaging as defined below, confirmed minimum of 1 year apart. Subsequent evaluations can be used to further specify the duration of negativity (e.g., MRD-negative at 5 years)
Flow MRD-negative	Absence of phenotypically aberrant clonal plasma cells by NGF on bone marrow aspirates using the EuroFlow standard operation procedure for MRD detection in multiple myeloma (or validated equivalent method) with a minimum sensitivity of 1 in 10^5^ nucleated cells or higher
Sequencing MRD-negative	Absence of clonal plasma cells by NGS on bone marrow aspirate in which presence of a clone is defined as less than two identical sequencing reads obtained after DNA sequencing of bone marrow aspirates using the LymphoSIGHT platform (or validated equivalent method) with a minimum sensitivity of 1 in 10^5^ nucleated cells or higher
Imaging positive MRD-negative	MRD negativity as defined by NGF or NGS plus disappearance of every area of increased tracer uptake found at baseline or a preceding PET/CT or decrease to less mediastinal blood pool SUV or decrease to less than that of surrounding normal tissue

*MRD, minimal residual disease; NGF, next generation flow; NGS, next generation sequencing; PET, Positron-emission tomography; CT, computed tomography; SUV, Standardized Uptake Value*.

According to IMWG criteria, MRD negativity might be detected within the BM, either by Multicolor Flow Cytometry (MFC) or by Next Generation Sequencing (NGS) technologies; this is unless sensitivity reaches at least 10^−5^, in which case it is defined as “sustained,” if confirmed minimum of 1 year apart (9).

Nevertheless, some technical issues are still open regarding the optimal assay to use to detect either phenotype (e.g., next generation multidimensional flow cytometry, imaging) or genotype aberrations (e.g., ASO-RQ PCR, digital droplet PCR, NGS) and their standardization, and the sample source (BM or peripheral blood, PB) and its pre-processing (red-cell lysis vs. Ficoll, fresh vs. frozen samples, requirement of CD138^+^ cells enrichment).

In this paper, we will review the more recent techniques developed to assess MRD in MM, their limits in sensitivity and applicability and their application in several clinical contexts.

## State-of-the-Art of Methods for Residual Myeloma Cells' Detection Within the Bone Marrow

Initially, MRD has been assessed on BM samples by amplifying the V(D)J clonal rearrangements, first just to gain qualitative information by polymerase chain reaction (PCR) (22, 27, 31) using clonal-size based methods (PAGE, GeneScanning) (17, 32), then by allelic specific oligonucleotide PCR (ASO-RQ-PCR), to also obtain quantitative information (22, 33, 34).

**Figure 2:**
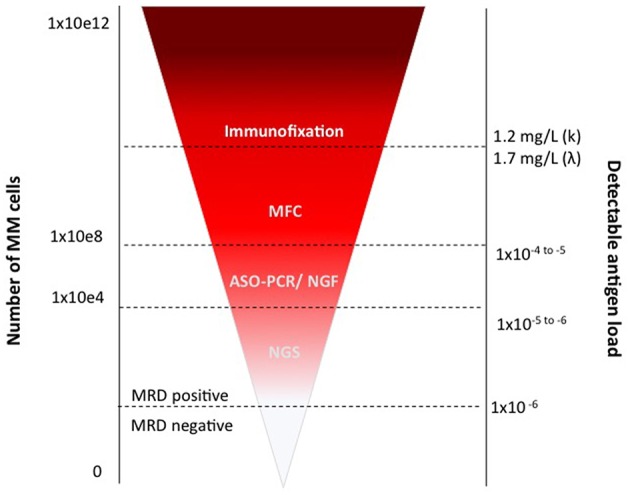
MRD assessment by MFC and PCR.

At present, modern technologies allow the detection of up to one on 10^4^, or on 10^5^, or even on 10^6^ cells, depending on their throughput. Both MFC approaches, which have been progressively improved up to the so-called Next Generation Flow (35, 36) (NGF), and NGS, which proved clear advantages over ASO-PCR, can detect very low levels of residual disease in the BM (Figure 2). These methods are actually almost superimposable, in terms of MRD detection power (13), supporting both the lack of unanimous preference for any specific technique, and the IMWG recommendation to choose one of the two on the basis of local availability (9, 37).

However, given the spatial heterogeneity and patchy nature of MM, MRD detection in BM might lead to false negative results, by underestimating the disease burden due to the presence of remaining, extra-medullary tumor cells. Indeed, despite a low rate of recurrence, MRD-neg patients can still relapse or develop extramedullary disease. Therefore, additional assessments to detect MRD in PB have been proposed, searching for circulating tumor cells (CTCs) by MFC or circulating free DNA (cfDNA) by NGS or including imaging techniques, such as PET and MRI to capture temporal and spatial genetic heterogeneity.

Due to the temporal heterogeneity of MM, the optimal time points of MRD detection have not been yet standardized. Most information arises from clinical trials enrolling newly diagnosed transplant-eligible MM patients, where MRD has been assessed after high-dose therapy. Nevertheless, since the genome is plastic, the amount of MRD could not be enough informative on the dynamics of disease evolution. Moreover, few data are available on systematic sequential analysis throughout the later stages of treatment; therefore, it is so far not clear how long patients should be followed during the disease course.

## Bone Marrow Hemodilution Is the Major Pitfall of Sample Collection for MRD Analysis

High throughput techniques require high amounts of starting material and sample pre-processing standardized protocols are required to optimize the procedure across laboratories, in order to limit the loss of biological material (38).

Since BM aspirates' hemodilution is the most common technical pitfall in MRD assessment (39), it is recommended to evaluate sample cellularity (e.g., by quantification of erythroblasts >5% and mastocytes by flow cytometry or smear) before proceeding with MRD evaluation, either by MFC or by NGS.

Overall, the red blood cell lysis (so-called bulk lysis) with ammonium chloride is considered the optimal pre-analytical procedure, as compared to the previously employed Ficoll-hypaque stratification (40).

The immune-magnetic CD138^+^cells enrichment, which is commonly performed at diagnosis in most MM patients for baseline FISH analysis, provides a concentrated source of neoplastic cells. This increases the possibility to detect the clonal marker by molecular approaches (33) and overcomes the issue of bad quality BM aspirates; on the contrary MFC methods require whole BM samples, to be able to describe PC in their cellular context.

Immunomagnetic separation might be replaced by flow-activated cell sorting (FACS), to warrant recovery of high-purity cells also from very low infiltrated BM aspirates, with the advantage to discriminate between normal and clonal PC sub-clones (3).

Whereas, molecular biology methods might be performed in batches on stored samples, MFC MRD assessment should be ideally performed within few hours from BM aspirates, since CD138 tends to be internalized by PCs. In addition, since the employment of anti-CD38 therapies might mask CD38 on PC surface, specific multiepitope CD38 antibodies need to be employed in patients treated with this kind of therapies (41).

## MRD Detection by Multiparametric Flow Cytometry (MFC)

The latest IMWG 2016 guidelines suggest using MFC techniques with EuroFlow standards to identify clonality and aberrant PC immune phenotype (9, 36, 42–44). The EuroFlow 8-color 2-tube method uses surface-only staining in 1 tube that consists of individually added antibodies and a separate tube that uses a 2-step procedure of surface staining followed by cytoplasmic light chain staining (36), since permeabilization of PCs can impair their light scatter properties and the expression of cell surface antigens (45). The expression of several markers discriminates normal from neoplastic PCs: CD138 is typically highly expressed by neoplastic PCs; CD38 and CD45 expression is lower in neoplastic, as compared to normal PCs; neoplastic PCs lack the expression of CD19, whereas they strongly express CD56 (46). Further markers (CD27, CD81, CD200, CD307, and CD117), might help to discriminate normal from neoplastic PCs and to identify phenotypes associated with certain outcomes (47, 48): if the detection of CD27, CD81, and CD117 is not included, there is the risk of overestimating the number of neoplastic PCs in any given sample by falsely including normal polyclonal terminal stage PCs (3, 44, 49, 50). Finally, PCs clonality may be assessed by evaluating the cytoplasmic immunoglobulin (CyIg)κ vs. CyIgλ expression (44, 50, 51).

Consensus guidelines suggest a lower limit of detection (LOD) for MRD in MM-BM of 0.001%, and ideally a limit of quantification of 0.001%, which requires at least 3 × 10^6^ and 5 × 10^6^ BM cells to be measured, respectively (44, 49, 50).

## Reproducibility of MFC-based Approaches for MRD Assessment

Despite the major advantages of MFC in MRD detection including, but not limited to, high-applicability, rapid turn-around time, intrinsic quality control check, a lack of requirement of patient baseline sample (differently from ASO-RT-PCR and NGS), and cost-effectiveness, there are several caveats for its applicability in clinics, including a lack of reproducibility and lower sensitivity compared to molecular techniques that identify patients with poor outcomes. Indeed, MFC-MRD detection provides limited molecular information about MM and, since performed on BM-PC, does not give any information about extramedullary disease (52).

The most common variations that can increase the lack of reproducibility among different groups include pre-processing (the quantity and quality of red-cells lysis buffer) and the use of fluorochromes conjugated to antibodies for each CD marker with consequent different staining index (15). Some groups, for example, prefer to avoid tandem dyes like APC-Cy7 and PE-Cy7, that can degrade due to sample processing (e.g., light, fixation, exposure to elevated temperatures) and, by emitting in the parent dye detector (APC or PE), might lead to a false positive count of events detectable in APC or PE channels. For this reason, the EuroFlow panel recommends the employment of APC-H7 (from BD Biosciences) instead of APC-Cy7.

An emerging issue in the field is the MRD monitoring in patients treated with anti-CD38 therapy, since the antigen masking. A group recently suggested modifying the EuroFlow panel using CD138 instead of CD38 for BM-PC detection, in the SRL-Flow panel (41). However, in SRL-Flow markers associated with bad outcomes, including CXCR4, CD200, and CD56 are omitted, and despite that the panel is cheaper than EuroFlow-NGF, it is not currently recommended for MRD detection.

Since published studies used a different panel design, fluorochrome conjugates, lysis reagents and sensitivity thresholds, caution is required while comparing MRD results deriving from longitudinally collected samples, as evaluated by different laboratories, especially if unstandardized assays have been employed: indeed very low levels of residual disease (e.g., 10^−5^ or 10^−6^) are prone to be improperly quantified (15, 41). For this reason, there are several attempts to standardize FCM to improve MRD detection as required by current and future clinical trials. In order to harmonize the use of any given instrument (e.g., FACSCantoII or Navios) to obtain reliable MRD values with at least 10^−4^ threshold (53), a further standardization process and technological improvement is needed; indeed still too much discrepancies are frequent at 2.5 × 10^−5^ threshold (53) or less.

To address the laboratories heterogeneity, in 2014 the College of American Pathologists (CAP) suggested the inclusion of new requirements, that each laboratory should include the limit of detection (LOD) or the lower limit of enumeration for flow-based MRD assays in the final diagnostic report, and to document how an MRD assay's LOD is measured (54). Similarly, the International Clinical Cytometry Society (ICCS), European Society for Clinical Cell Analysis (ESCCA), and the Euroflow Consortium recommend the harmonized use of different reagents, fluorochromes panels, sample processing, platforms, LOD and data analysis, to improve the accuracy, sensitivity and specificity of MFC-MRD detection in MM (15, 44, 50).

## Open Issues of MFC-based Technologies for MRD Assessment

Several questions concerning the MFC-based MRD assessment are still under investigation. First, the optimal sample source for MRD detection (BM vs. PB). Second, the frequency of assessment and its longitudinal development, which usually includes post-induction, third and 24th month post-transplant. Third, the setting of patients, as most studies involved patients evaluated after first induction and at different timepoints (within 24 months) post-transplant (55, 56). As expected, consolidation and maintenance can further improve the depth of response and the frequency of MRD negativity (23, 24, 56–58). However, patients, who achieved MRD negativity sooner, have better outcomes, as compared to those who became MRD-neg later, after ASCT (28). Low MRD levels correlate to longer PFS, with progressive MRD log-reduction at different timepoints (after either induction, single or double ASCT) (55, 57). However, controversial results have been reported in patients with unfavorable cytogenetic risk, who might either benefit (57–59) or not (46) from MRD negativity achievement (Supplementary Table 1) (60), even if these results might be the consequence of different time of MRD assessment. Indeed, in the MRC (Medical Research Council) Myeloma IX trial, MFC-MRD negativity, as evaluated at day 100 after ASCT, but not after induction, predicted for longer PFS (58). Of interest, the same study showed that approximately 1-year of median OS benefit might be ascertained to each log of MRD level reduction (61).

**Figure 3:**
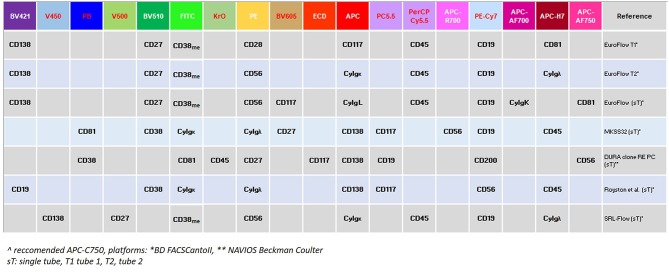
6-8-10 color panels used in MFC/MRD detection.

Fourth, technological efforts are still required to discriminate normal from neoplastic PCs, with a sensitivity of at least 10^−5^. Indeed, MRD levels between 10^−4^ and 10^−5^ and lower than 10^−4^, both equally and favorably affect patients' outcome (26, 61). The introduction of second and third generation MFC technologies, moving from 4- to 8-, or even to 10-colors panel might considerably improve the sensitivity of MRD evaluation (26, 61). Figure 3 summarizes the 9-10-colors, second-generation MCF panels, more frequently employed in the context of large clinical studies (e.g., clinical studies and/or retrospective real-life surveys), which might even be implemented in the daily clinical practice (41, 42, 62, 63).

## MRD Detection by Conventional Molecular Approaches: ASO-PCR and qPCR

Polymerase Chain Reaction (PCR)-based MRD detection identifies persistent tumor cells through the amplification of the clonal immunoglobulin heavy-chain gene variable region (VDJ-IgH) gene rearrangement. In the ASO-PCR approach, an allele-specific oligonucleotide (ASO) primer, complementary to a highly variable region such as IgVH (complementary determinant regions, CDR) associated to a consensus primer (frequently located within the joining region) improved feasibility of the approach, achieving good sensitivity (nearly 1 × 10^−5^). Nowadays, ASO-PCR is by far the most widely used technique, either in a qualitative (22) or in a quantitative way, if primers are coupled to a fluorescent probe to monitor the amplification in real-time assays (qRT-PCR) (64, 65).

The sensitivity of MRD detection of quantitative polymerase chain reaction (qPCR) approach depends on several factors, including the type of VDJ rearrangement, the dimension and specificity of the junctional region and the amount of DNA available for each reaction. Since MRD levels can be reliably quantified when a relatively high number of pathological cells is present, hemodilution or insufficient quantity of PCs in the sample can limit the accuracy and feasibility of the detection itself.

The high level of somatic hypermutations within the IgH-CDR, both by causing variable levels of primer annealing of consensus primers and by impairing the clonal detection with unpredictable amplification and quantitation of results, limits ASO-PCR applicability to almost half of patients (23, 64, 66, 67). However, with a better primer and probe selection (66), or choosing targets other than VDJ-IgH such as kappa deleted region (KDE) (67), it is possible to improve specificity, sensitivity and applicability of ASO-PCR (Table 2). Overall, lack of clonality detection, unsuccessful sequencing and suboptimal PCR amplification are the most common drawbacks (64, 68).

**Table 2:** Comparison between different techniques to detect MRD in MM-BM.

	**Multidimensional flow cytometry (phenotype detection)**					**Molecular techniques (genotype detection)**	
	**First generation**	**Second and third generation**				**Second generation**	**Third generation**
	**2008 EMN consensus (4–6 colors)**	**Euroflow (6 colors)**	**2016 ICCS consensus (8 or more colors)**	**DURAClone RE PC (9 colors)**	**MKSSC32 (10 colors)**	**ASO-qRT-PCR**	**NGS**
Number of cells required		20 × 10^6^ leukocytes	2–5 × 10^6^ leukocytes	5 × 10^6^ leukocytes	20 × 10^6^ leukocytes	500 ng, 1 × 106 PCs for triplicate analysis	1,400 ng, 2 × 106 PCs for triplicate analysis
Theoretical LOD/LOQ	4 × 10e^−3^	4 × 10e^−5^/4 × 10e^−6^	4 × 10e^−4^	8 × 10e^−6^ 2 × 10e^−5^	2 × 10e^−5^ (10 × 10^6^ cells staining capacity)	10e^−4^	10e^−5^
Applicability (% cases)	95	99	95	99	99	50–90	80–90
Pre-treatment evaluation and sample quality assurance	Required	Not required	Not required	Not required	Not required	Required	Required
Required sample at diagnosis	NO	NO	NO	NO	NO	YES	YES
Required fresh sample	YES	YES	YES	YES	YES	NO	NO
Turnaround	60–90 min	60–90 min	60–90 min	60–90 min	60–90 min	days	days
Cost	**+**	**++**	**++**	**++**	**++**	**+**	**++++**
Availability	Widely available	Specialized labs				Intermediate	Specialized labs
Harmonization	YES (EMN)	YES (EMN)	YES (ICCS/ESCCA)		Ongoing	YES	NO

*EMN, European Myeloma Network; ASO-qRT-PCR, allele-specific oligonucleotide-quantitative polymerase chain reaction; PC, plasma cells; min, minutes; LOD, limit of detection; LOQ, limit of quantitation*.

Other qRT-PCR, different from IgH variable regions, such as FGFR3, have been used in detection of MRD in MM with promising results (69). Alternatively, the use of digital droplet PCR (ddPCR) has been proposed and validated to evaluate MRD in MM (70, 71) with results that were comparable to those obtained with qRT-ASO-PCR and a greater applicability (71), since in ddPCR a reference standard curve is not required to quantitate the disease-related transcript.

## MRD Detection by Next Generation Sequencing (NGS)

Next Generation Sequencing (NGS) technology allows the processing of millions of sequence-reads in parallel, making this high throughput the perfect tool for the development of NGS-based assays for MRD detection. In fact, this technology is not just used for covering broad genomic regions, but also for the ultra-deep sequencing of small genomic regions, thus resulting particularly convenient also for highly sensitive detection of the MM-associated clonal rearrangements of the IgH gene, the marker for molecular MRD evaluation. Indeed, the wide complexity of the IgH gene locus on chromosome 14q32 is much more manageable by NGS approaches, as compared to the conventional Sanger sequencing (Table 2). In addition, NGS can further simultaneously detect other Ig clonal markers, e.g., those deriving from light chain rearrangements.

The most common NGS approach for Ig sequencing is amplicon-based: it employs sets of multiple primers targeting IgH-VDJH, IgHV-leader regions, IgH-DJH and Igk rearrangements. Over the years, lot of efforts have been devoted to optimizing primer design, as compared to the originally employed BIOMED2 (72) ones, in order to minimize off-target sequencing, which might represent a major issue of deep sequencing experiments. Currently available most employed primer sets are mainly included in commercial kits and their exact genomic location has not been released; the set-up of a more efficient Ig NGS assay is currently also the goal of the EuroClonality NGS consortium (www.EuroClonalityNGS.org), but technical details have not yet been reported. After amplification, the Ig library is deeply sequenced, to determine the frequencies of myeloma-specific clone(s).

Another possible NGS approach is a capture-based assay: it employs a hybridization panel of probes covering all coding V, D and J genes of the Ig locus (73). Even if theoretically this approach might provide a more realistic representation of the Ig rearrangement, not affected by PCR amplification bias, it has been less experimented, and few data are so far available with this approach.

As for ASO-PCR, also for NGS approach the Ig detection requires two subsequent main experimental phases: (1) the so-called screening (or clonality ID test), aimed at the definition of the patient-specific clonal rearrangement(s) characterizing patients at diagnosis and (2) the actual MRD evaluation in follow-up samples with low tumor burden.

## Pitfalls of NGS-based Approaches for MRD Assessment

The screening phase mostly benefit from the application of NGS, since the nature itself of NGS data, by providing both qualitative and quantitative information, allows accurate and sensitive estimation of clonal size. By employing sequence alignment bio-informatic tools, the frequency of identical sequence can be easily assessed and clonality can be defined, commonly by setting an arbitrary threshold at 5% frequency. This allows the definition of a patient-specific marker in virtually all newly diagnosed MM cases (74–76). In practice, even if the primer sets cover as wider as possible the Ig locus, the Ig somatic hypermutation within the targeted primer-binding sites still might remain the major pitfall, by preventing the primer to bind effectively in a small subset of myelomas. Another major issue at diagnosis is represented by sample hemodilution, which might impair the detection of the clonal rearrangements, as well.

On the other hand, even if in theory the MRD evaluation in follow-up samples would be simplified by NGS technology, several pitfalls still raise, mainly concerning the bio-informatic analysis of sequencing data, as obtained in samples with very low tumor burden. The goal is to recover the exact clonal sequence as detected at diagnosis in the follow-up samples, where it might be present at very low frequency. NGS-based approaches are supposed to detect MRD levels below 10^−5^ (i.e., less than one myeloma cells out of 100,000 normal cells). Nevertheless, since low frequency sequences needs to be distinguished from the noise of the experimental background, in order to be as much statistically confident as possible in detecting the MRD sequence, enough input DNA needs to be deep enough sequenced. To sequence a high number of samples, multiple libraries are required, which is technically challenging and, more importantly, quite expansive. The implementation of some technical aspects (synthetic immune repertoire as internal control and optimized PCR primers minimizing the impact of somatic hyper-mutation on Ig amplification) and the use of bio-informatic expedients, both represent the peculiarity of the most commonly employed, commercially available NGS kit for MRD assessment in MM (clonoSEQ®, Adaptive Biotechnology), which has actually overcome most of the technical pitfalls of MRD evaluation by NGS. By employing the above-mentioned kit, MRD results are commonly reported as “<10^−6^,” “from 10^−6^ to <10^−5^,” “from 10^−5^ to <10^−4^,” and “10^−4^ or greater” (https://www.clonoseq.com/now-fda-cleared).

Despite its high performances, costs of this technology are very high, and therefore inadequate for daily clinical patients' management and even if no specific MRD recommendations have been so far included in any international guidelines for the daily management of MM patients, it might be predictable that such recommendations will be suggested soon. Therefore, more affordable NGS assays should be implemented in the clinical practice, either by forsaking the objective of 10^−6^ sensitivity in all evaluations, or by assessing MRD by less expansive, yet highly sensitive technologies, such as ddPCR. This latter possibility might be more realistic and easily applicable in the near future.

## The Clinical Setting: ASO-PCR vs. MFC

The applicability of MFC-MRD might be widely extended to most MM patients, based on the use of standard disease-associated markers. In contrast, quantitative real-time PCR approaches are less applicable, basically due to their patient-specific features. In addition, flow-MRD incorporate a quality check for early identification of hemodilution in BM samples, thus reducing the risk of false-negative MRD results. Thanks to an increased sensitivity and the reduction of the “limits of detection” (LOD) and of the “limits of quantification” (LLOQ), flow-MRD is directly quantitative, whereas qPCR approaches, as being calibrated to a standard curve, commonly have variable LOD/LLOQ, according to IGHV variable region gene sequence (49). However, in few studies, which compared side-by-side MCF and qPCR, any clear advantage of one method over the other has been highlighted.

A comparison of NGS, MFC and ASO-PCR MRD methods has been carried out in 133 MM patients in at least very good partial response (VGPR) after front-line therapy, overall showing high concordance between the MCF and ASO-PCR. A longer time to tumor progression and overall survival were observed in NGS-MRD-neg patients, as compared to those who were MRD-pos, with the larger advantage in patients with a deeper response, if patients were stratified by different levels of MRD (74).

In a smaller study, including 22 MM patients in at least VGPR after VD-based induction followed by ASCT at different timepoints, ASO-PCR and MFC identified MRD negativity with 75% concordance and both predict PFS at 48 months. Similarly, MRD negativity assessment by ASO-RQ-PCR (sensitivity, 10^−5^) and MFC (sensitivity, from 10^−4^ to 10^−5^) was highly concordant in 73 patients from the RV-MM-EMN-441 and RV-MM-COOP-0556 phase 3 trials who achieved at least a VGPR after intensification/consolidation (24).

In 170 patients enrolled in three consecutive Spanish trials achieving at least partial response after treatment, MFC and ASO-RQ PCR showed a significant correlation in MRD quantitation. Also, in this study, patients with <10^−4^ residual tumor cells showed longer PFS compared with the rest. Among patients who achieved at least VGPR, PCR discriminated two risk groups with different PFS and OS (64). Due to high technical demands, some authors suggest reserving ASO RQ-PCR to patients in immunophenotypic remission (66). However, in the pooled analysis of three large clinical trials of the PETHEMA/GEM group, including 609 patients eligible or not to ASCT, patients who were MRD-neg despite a persistent M-component showed similar PFS and OS to patients with MRD-neg disease in CR (19). This counterintuitive observation might be ascribed to late serologic responders or false negatives by MFC, due to persistent clonal PCs outside the BM or in a BM area for which the sample obtained was not representative (14). Consequently, MRD should be tested also in VGPR patients, since the clearance of monoclonal component does not overlap with MRD status (14, 28, 56, 58). On the other hand, MRD-negativity surpassed the impact of CR on PFS and OS across the disease spectrum, regardless of the type of treatment or patient risk group (77). In 460 myeloma patients, who achieved CR and MRD negativity by MFC, MRD status could predict response durability without any improvement in OS, probably for the need of standardization at time of assessment (78) and requirement of lower LOD.

Taken together, studies in the last years suggest that ASO-PCR can reach a similar if not even superior sensitivity MFC (23, 24, 64, 66, 70). Results are usually superimposable, especially in MRD-pos cases, while in few samples, MRD-neg by MFC, it is possible to find a PCR positivity (66) and when it is possible reaching LOD 10^−5^ (24, 79). Low MRD levels obtained by the molecular methods reported were always associated with a better long term PFS and demonstrated that innovative (targeted) drugs may reduce the tumor burden at unprecedentedly reached very low, or even undetectable, levels (22). One of the PCR-based methods advantages over MFC is that it allows the use of DNA extracted from archival samples (such as BM smears), widening the possibility to search for MRD in retrospective studies, or when fresh or cryopreserved cells are not available. Indeed, using archival material it has been possible to design clonotype-specific primers in 60% of cases (80).

## NGS in the Clinical Setting

In MM, MRD has been evaluated by NGS mainly in the context of clinical trials and most available data have been produced using the clonoSEQ® platform, previously known as LymphoSight® platform (Sequenta Inc., later acquired by Adaptive Biotechnologies) (70, 74, 81). This technology, which has been recently received the FDA approval for marketing (82), is highly performant both for the definition of clonal rearrangement at diagnosis (so called Clonality ID test) and for a sensitive and specific MRD evaluation (10^−6^ guaranteed sensitivity), thus perfectly responding to the clinical research need for early regulatory surrogate end-points for drug approval.

Therefore, until now, only a few groups explored the clinical relevance of MRD negativity by NGS, especially in patients who received monoclonal antibodies as part of their treatment.

In the IFM DFCI trial, exploring the role of ASCT and maintenance in newly diagnosed myeloma patients (224 of 366 patients at the start and 183 of 239 patients after completing maintenance therapy) treated with lenalidomide, bortezomib, and dexamethasone (RVD), NGS-MRD negativity (<10^−6^) showed the importance of response depth in longer PFS and OS (83). Differently from similar studies based on ASO-RT-PCR, survival analyses, limited to the population of patients assessed before and after maintenance therapy, showed a similar PFS and OS either for patients who maintained MRD negativity at both measurements, or for those who became MRD-neg after 12 months of maintenance (83). This could be due to the higher sensitivity of NGS in identifying MRD-neg patients or to the treatment itself. Moreover, even using lower LOD there is still a significant proportion of patients that relapse despite MRD-neg achievement, highlighting the high complexity of MM biology.

In the PETHEMA experience, the pooled analysis of NDMM patients who achieved at least VGPR in the GEM 2000 and GEM05MENOS65 trials, molecular response by deep sequencing (corresponding to MRD negativity with the sensitivity <10^−5^), was associated with significantly longer TTP and was the single variable with statistical significance in the multivariate model for TTP (74).

In 45 NDMM patients treated up-front with carfilzomib and lenalidomide-based (KRd) induction followed by lenalidomide maintenance confirmed the high-rate of MRD negativity achievement (84), associated to longer PFS, also in a recent update presented in abstract form (85). Emerging data from the same group show that KRd therapy takes 6 cycles to achieve MRD negativity (86).

In the setting of elderly newly diagnosed MM patients enrolled in the ALCYONE trial, there was >3-fold higher MRD-negativity rate when daratumumab to VMP as a single agent as part of maintenance, as presented by Dr. Mateos at the last ASH annual meeting (87). Similar results have been obtained in the POLLUX and CASTOR trials, in which daratumumab combined with standard of care improved MRD-neg rates (88–90).

Since continuous improvement of sequencing technologies will most likely reduce costs and processing times, NGS could become soon a viable option for routine clinical practice for prognostication and MRD detection in MM (91). In addition, patients could also be stratified based on mutational load or neoantigen load (92), with the advantage to look for new mutations, with distinct effects on survival and tailored therapy for each individual patient (8).

## Future Directions in MRD Assessment: Within the BM Only?

MRD is recognized as the most important prognostic factor in MM, independent from ISS disease stage, therapy, high-risk cytogenetics (16, 30, 56), methodology (if able to reach a sensitivity of at least 10^−5^). Some authors have proposed MRD as a surrogate of outcome (PFS and OS) and a new endpoint to achieve in MM treatment (93), at least in clinical trials (77, 94), due to the long-term treatment and increased quantity of high-quality responses with second and third-generation of novel agents. On the contrary, others consider MRD as prognostic factor for transplant-eligible newly diagnosed MM patients (95), worth of standardization and harmonization before to be largely applied in real life. Since currently available data have shown prognostic impact but not correlation between MRD and outcome, due to heterogeneous datasets (study population, different treatments, different assays, and cut-off), regulatory agencies still consider MRD as an intermediate and not main endpoint for ongoing and future clinical trials. Moreover, the heterogeneity of MM clinical course and patients (e.g., newly diagnosed vs. relapsed/refractory, transplant eligible vs. frail) and clinical costs suggest individualizing the MRD assessment (95). To overcome these and other emerging pitfalls in the wide applicability of MRD in clinical practice there are several strategies: liquid biopsy, enumerating circulating tumor cells by MCF and the so-called immune positron emission tomography (PET).

## Liquid Biopsy and NGS in Peripheral Blood

NGS of IgH gene rearrangements is highly sensitive and allows the identification of small residual sub-clonal populations throughout the therapeutic treatment, which is not always possible with MFC or PCR. However, the patchy pattern of BM infiltration of MM can affect MRD-neg results, irrespectively of the technique adopted.

Recently it has been suggested that the so-called “liquid biopsy” might overcome this issue. Liquid biopsy is a non-invasive strategy for monitoring disease dynamics through the analysis of circulating cell-free tumor DNA (ctDNA), which is the fraction of total cell-free DNA (cfDNA) fragments circulating in the bloodstream that are derived from cancer cells. By monitoring ctDNA, liquid biopsy might provide information regarding the genetic landscape of cancer (96).

The role of liquid biopsy as a tool to detect residual cells in PB of myeloma patients has been first evaluated by two pilot studies, interrogating ctDNA by NGS (72, 97). Both studies aimed at investigating the IgH gene rearrangement within ctDNA, by means of an amplicon-based NGS approach based on BIOMED2-FR1/-FR3 (IgH), -Ig kappa (IGk) or -Ig lambda (IGl) primer pools, both at diagnosis and after treatment. This approach proved quite successful at diagnosis, with almost 100% of cases characterized, whereas it resulted overall controversial on samples collected from patients in CR. Indeed, while Oberle et al. showed a high rate of failure in IgH rearrangement detection on ctDNA samples collected from patients in CR (18/27: 66% of samples) (72), Biancon et al. was able to monitor the disease in three time point throughout therapeutic treatment in all patients, both in the BM aspirated and in the ctDNA, with a sensitivity of <1 on 10^5^ cells (97). In addition, authors demonstrated a high correlation between the disease detection at study entry by 8-color MFC and by ctDNA and the overall amount of ctDNA at diagnosis was correlated to worse prognosis in term of PFS (97).

More recently, another group explored the possibility to use ctDNA to monitor MM disease by adopting the commercially available clonoSEQ® NGS kit for MRD assessment (Adaptive Biotechnology) (98), yet still showing very low consistency between results, as obtained from paired plasma and BM samples (49%), especially in patients with very low disease burden, resulting MRD-pos with BM and MRD-neg with ctDNA samples. Therefore, with a negative predictive value of 36% and a positive predictive value of 89%, authors stated that any correlation between ctDNA and BM for MRD by IgH NGS-only might be highlighted in MM patients, thus suggesting an overall inefficacy of this approach, as compared to the standard one, to monitor MRD in MM patients (96). However, this experimental plan did not take into account that residual disease clearance might have different dynamics in the liquid biopsy, as compared to the BM (99), thus impairing the possibility to monitor residual disease reduction in PB by the same timepoints as for MRD evaluation in the BM. Moreover, other technical pitfalls concern the employed NGS amplicon strategy, which targets DNA fragments longer than ctDNA ones (on average around 160–180 bp long): indeed, slightly different NGS strategies might be more sensitive, by targeting shorter amplicons and by increasing the input amount of ctDNA as well.

Overall, the possibility to monitor residual disease dynamics by alternative, non-invasive assays such as liquid biopsy, even if not yet fully exploited in MM, still seems relevant enough to justify more efforts in solving technical issues. Indeed, it has been demonstrated that, since ctDNA—by deriving from different tumor cells—might recapitulate the genomic landscape of tumor itself, the detection and monitoring of single nucleotide variants (SNVs) in ctDNA might allow to extensively monitor disease dynamics after therapy also in MM patients (99–101). This approach proved even more sensitivity when more than one target is monitored and analyzed with specific bio-informatic tools, able to improve sensitivity for minute quantities of residual disease (102). Therefore, once validated, the use of ctDNA, either targeting IgH rearrangements and/or SNVs, might be integrated in the conventional algorithm for MM monitoring, together with indirect immunobiochemical markers (i.e., monoclonal protein) and imaging techniques (such as PET-CT or WB-MRI), thus possibly contributing to the utmost definition of residual disease dynamics in MM.

## Circulating Tumor Cells in Peripheral Blood and Future Directions

Besides ctDNA, liquid biopsy conventionally includes circulating tumor cells (CTCs), which might be released from BM to PB due to several, not always recognized reasons (100). Biological mechanisms inducing PCs to circulate might involve an increasing independence from adhesion to the niche, suggesting an association between the increment of CTCs and both the risk of malignant transformation from MGUS to SMM to symptomatic MM, and an inferior survival of symptomatic newly-diagnosed and relapse/refractory MM with high levels of CTCs (103, 104), as recently reviewed (105).

MFC might be efficiently employed to analyze CTC in MM. By MFC, it has been shown that circulating neoplastic PCs are mostly quiescent (arrested in the subG0-G1 phase of the cell cycle), with a proliferation index (percentage of cells in S-phase) significantly lower, as compared to that of their BM counterpart, though with a peculiar clonogenic potential, therefore possibly representing a unique subset of patient-paired BM clonal PC (104, 106).

Under physiological conditions, long-living BM PCs are not driven to circulate in PB; conversely, CTCs are increased in monoclonal gammopathies, following a circadian rhythm similar to CD34+ cells (104). The downregulation of integrins (CD11a/CD11c/CD29/CD49d/CD49e), adhesion (CD33/CD56/CD117/CD138), and activation molecules (CD28/CD38/CD81) typically characterizes circulating neoplastic PCs (104, 107).

Recently, an automated assay (CELLSEARCH®, Menarini Silicon Biosystems Inc.) has been developed, able to isolate and enumerate the CTCs from the PB of MM patients, based on the expression of specific antigens (108). This technology showed that in newly diagnosed MM, CTCs count correlated with other clinical measures of disease burden at baseline and that it was reduced after effective treatment.

Like ctDNA, CTCs might provide a simultaneous picture of both medullary and extramedullary disease (106). Indeed, in CR patients, MRD evaluation by employing two techniques (MFC and imaging, either PET or MRI) proved a persistence of spatially separated clones with residual focal lesions, detectable in half of first-line patients, associated with shorter PFS; therefore, double-negative and double-positive features might be observed, associated with very good and bad PFS, respectively (109).

CTCs might also be analyzed by molecular approaches (106, 110, 111) and therefore employed as biomarker of response to therapy (72, 112).

Overall, by mirroring the entire heterogeneity of the tumor, both ctDNA and CTCs might be used as diagnostic markers and represent a promise for MM monitoring: being a non-invasive procedure, liquid biopsy can provide a quick read-out of the disease state and might complete the molecular profiling of the tumor, as evaluated on BM PCs.

For the same reason, several groups are investigating imaging as further tool for MRD detection out of BM. An interesting technique could be the so-called immune-PET, which detects targeting of specific antigen by therapeutic antibodies, combining monoclonal antibodies and positron- to achieve optimal tumor-to-background activity ratios, as recently reviewed (113). Even if never tested in MM, this approach has been shown feasible in metastatic breast cancer candidates to trastuzumab emtansine (114). For example, an anti-CD38 or anti-BCMA immuno-PET would allow measurement of target expression also in extramedullary lesions, limiting misinterpretation due to spatial heterogeneity and sub-clones branching.

## Conclusions

Over the past 15 years, MM treatment has improved, thanks to the progressive availability of novel classes of agents targeting MM sub-clones in their BM microenvironment. These highly active drugs, combined with each other, have determined a dramatic increase in the rate and depth of response, up to the level of MRD-negativity, as detected within “single-site” BM aspirates, tested by highly performant MFC and/or molecular biology techniques.

MRD negativity is currently considered as the most powerful predictor of favorable long-term outcomes in MM and is likely to represent the major driver of treatment strategies in the near future.

Therefore, intense research is currently focused both on improving the currently employed technology and on exploring alternative methods for MRD testing in MM, major goal being the development of easy, frequently repeatable and reliable techniques, possibly able to recapitulate intra-clonal and spatial heterogeneities characterizing MM, as well. Among others, liquid biopsy represents one a most promising strategy to overcome the shortcoming of BM sampling, potentially coupled with imaging methods, in order to get the most holistic image of the disease distribution, by exploring the disease dynamics both inside and outside the BM.

## Author Contributions

AR and CT designed the study and wrote the paper. GP reviewed novelties about ASO-RT-PCR. NP reviewed novelties about MFC. MM reviewed novelties about NGS and liquid biopsy. CC reviewed clinical utility of MRD detection. All authors read and approved the final version of the manuscript.

### Conflict of Interest Statement

The authors declare that the research was conducted in the absence of any commercial or financial relationships that could be construed as a potential conflict of interest.
