# Supplementary material for: Minimal Residual Disease Assessment Within the Bone Marrow of Multiple Myeloma: A Review of Caveats, Clinical Significance and Future Perspectives
Source: Front Oncol. 2019 Aug 20;9:699. doi: 10.3389/fonc.2019.00699 (PMC6710454; doi:10.3389/fonc.2019.00699)
Supplement: Supplementary file 1 [file Data_Sheet_1.PDF]

Table 1

IMWG criteria of response for patients with MM

Response SubCategory	Response Criteria
**sustained MRD-negative**	MRD negativity in the marrow (NGF or NGS, or both) and by imaging as defined below, confirmed minimum of 1 year apart. Subsequent evaluations can be used to further specify the duration of negativity (eg, MRD-negative at 5 years)
**Flow MRD-negative**	Absence of phenotypically aberrant clonal plasma cells by NGF on bone marrow aspirates using the EuroFlow standard operation procedure for MRD detection in multiple myeloma (or validated equivalent method) with a minimum sensitivity of 1 in 10^5 nucleated cells or higher
**Sequencing MRD-negative**	Absence of clonal plasma cells by NGS on bone marrow aspirate in which presence of a clone is defined as less than two identical sequencing reads obtained after DNA sequencing of bone marrow aspirates using the LymphoSIGHT platform (or validated equivalent method) with a minimum sensitivity of 1 in 10^5 nucleated cells or higher
**Imaging positive MRD-negative**	MRD negativity as defined by NGF or NGS plus disappearance of every area of increased tracer uptake found at baseline or a preceding PET/CT or decrease to less mediastinal blood pool SUV or decrease to less than that of surrounding normal tissue

Table 2 Comparison between different techniques to detect MRD in MM-BM

	**Multidimensional flow cytometry (phenotype detection)**					**Molecular techniques (genotype detection)**	
	**First generation**	**Second generation and third generation**				**Second generation**	**Third generation**
	**2008 EMN consensus (4-6 colors)**	**Euroflow (6 colors)**	**2016 ICCS consensus (8 or more colors)**	**DURAClone RE PC (9 colors)**	**MKSSC32 (10 colors)**	ASO-qRT-PCR	Next generation sequencing
**Number of cells required**		20×10^6 leukocytes	$2\text{-}5 \times 10^6$ leukocytes	5×10^6 leukocytes	20×10^6 leukocytes	500 ng, 1× 106 PCs for triplicate analysis	1400 ng, 2× 106 PCs for triplicate analysis
**Theoretical LOD/LOQ**	0.004	$4 \times 10^6/1 \times 10^5$	0.0004	$8 \times 10^6/2 \times 10^5$	$2 \times 10^6/5 \times 10^6$ (10 $\times 10^6$ cells staining capacity)	0.0001	0.00001
**Applicability (% cases)**	95	99	95	99	99	50-90	80-90
**Pre-treatment evaluation ansample quality assurance**	Required	Not required	Not required	Not required	Not required	Required	Required
**Required sample at diagnosis**	No	No	No	No	No	Yes	Yes
**Required fresh sample**	Yes	Yes	Yes	Yes	Yes	No	No
**Turnaround**	60-90 min	60-90 min	60-90 min	60-90 min	60-90 min	Days	Days
**Cost**	+	++	++	++	++	+	++++
**Availability**	Widely available	Specialised labs				Intermediate	Specialised labs
**Harmonization**	Yes (EMN)	Yes (EMN)	Yes (ICCS/ESCCA)		ongoing	Yes	ongoing (EuroMRD)

Supplementary Table 1
Impact of MFC/MRD status on cytogenetic risk

Study	Method, LOD	Outcome in MRD$^+$patients			
		PFS in high-risk cytogenetics	PFS in standard-risk cytogenetics	OS in high-risk cytogenetics	OS in standard-risk cytogenetics
Hu, 2019[60]	MFC, 10^{-4}	mPFS 19 mo	mPFS 24 mo	not reported	not reported
Li, 2019[14]	MFC, 10^{-4}	mPFS 22 mo	mPFS 34 mo	4-yrs OS 54%	4-yrs OS 84%
Chakraborty, 2017[59]	MFC, 10^{-4}-10^{-5}	mPFS 22 mo (in del17p pts)	not reported	mOS 50 mo (in del17p pts)	not reported
Paiva, 2012[107]	MFC, 10^{-4}-10^{-5}	3-yrs TTP 0%	3-yrs TTP 32%	3-yrs OS 57%	3-yrs OS 90%
Rawstron, 2013[58]	MFC, 10^{-4}	mPFS 8.7 mo	mPFS 33.7 mo	not reported	not reported
Liu, 2012[115]	MFC, 10^{-5}	mPFS 13.2 mo	mPFS 22.6 mo	mOS 22.7 mo	mOS 18.2 mo
Paiva, 2016[26]	MFC, 10^{-5}	mTTP 12 mo	mTTP 15 mo	median OS not reached	median OS not reached

Abbreviations: MFC: multiparametric flow cytometry; mPFS: median progression free survival; mOS: median overall survival; mTTP: median time to progression; mo: months.
